# Entropy as the main justification for research in medical ethics

**DOI:** 10.1186/s13010-022-00121-5

**Published:** 2022-05-04

**Authors:** Alban Zarzavadjian Le Bian, Louis Pantel, Christophe Tresallet, Marie-France Mamzer

**Affiliations:** 1 Service de Chirurgie Générale et Digestive, Hôpital Louis Pasteur, Hôpitaux de Chartres, 4, rue Claude Bernard, 28630 Le Coudray, France; 2 Centre de Recherche des Cordeliers, INSERM, Sorbonne Université, USPC, Université Paris Descartes, Université Paris Diderot, Équipe d’accueil ETRES, Paris, France; 3 Service de Chirurgie Générale et Digestive, Hôpital Avicenne, Assistance Publique – Hôpitaux de Paris, Université Paris XIII, Bobigny, France

**Keywords:** Medical ethics, Surgery, Research in medical ethics

## Abstract

Ethics is an unconventional field of research for a surgeon, as ethics in surgery owns several specificities and surgery is considered an aggressive specialty. Therefore, the interest of research in medical ethics is sometimes unclear.

In this short essay, we discussed the interest of research in medical ethics using a comparison to thermodynamics and mainly, entropy. During the transformation of a figure from one state to another, some energy is released or absorbed; yet, a part of this energy is wasted because of “unordered” (and unsuccessful) reactions: it is Entropy.

This “wasted energy” exists in Medical practice and justifies research in Medical ethics.

Surgery has always been one of the rare forgone conclusions in my life. As a young child, I realized the wonderful power of this discipline: my arm was shattered, crippled, excruciating, massively infected, and, when amputation was at some point mentioned, Surgery (and the amazing adaptability of life) made it functional again. Beyond outcomes, I perceived the mystical pattern of this profession: in case of a life-threatening, hopeless condition, the surgeon’s hands may cure, like a shaman’s or a prophet’s hands. And as a patient, I discerned how intimate the relationship with the surgeon was, the given trust enabling you to accept iron tools in your vulnerable body. Despite my young age, I knew that Surgery was more a matter of soul than flesh. From fascination to vocation, years and years after, I have become a surgeon. Ethics is certainly the counterpart of this deeply-rooted mission; I was a patient, I developed into a surgeon, and as I was seeking the fair act for patients (humans with diseases), I understood my inclination for Ethics. Therefore, I have learned Ethics as I have learned Surgery - daily, humbly, seriously – and researched in both fields.

Ethics is often considered an unconventional field of research for a surgeon. Because of the inner nature of Surgery – the consented violence, the *huis clos* – and the tormented relationship between Surgery and Medicine – Surgery was not regarded as a medical or academic specialty for centuries and was therefore left to butchers –, surgeons may be seen yet as immoral, bestial, bloodthirsty health workers, a nature that is discordant with ethical principles. This is obviously wrong and specific subjects of surgical ethics have been defined and studied (the unpredictability, the irreversibility, the variability, and the Genesis of evidence). Also, the layman might consider the ethician as a conceited Mr. Know-it-all: most imagine that Ethics draws the fine line between Good and Evil (when ethicians know that Ethics begins far beyond this line). Due to these potential reasons among others, I was recently requested to publicly justify Medical Ethics as a chosen field of research. I was speechless and surprised: I see Ethics in Medicine like Ecology in Politics, it should be implemented in all programs. Yet, I was unable to explain the obvious interest of Ethics in Surgery, making Ethics (and my research) useless for the auditory.

A couple of days later, with a persistent thought of my failure, I had an unexpected and brief moment of clarity. Packing useless papers at home, I found old lessons of chemistry from my very first year as a student in medicine: Thermodynamics. And as principles of thermodynamics were coming back to my mind, the shape of my justification appeared.

Thermodynamics is the field of science that describes, quantifies, and analyzes the transformation of a figure from one state to another; for example, from liquid to gas, or from “cold” to “hot” water. This change releases or absorbs energy (mostly heat). Yet, a part of this energy is wasted during the process because of “unordered” (and unsuccessful) reactions; the quantity of lost energy depends on the reaction, volume, and other factors, but is unpreventable: it is Entropy. As in Thermodynamics, the medical practice aims to change a figure (“the patient”) from a state (“unhealthy”) to another state (“healthy”). You can consider any definition for “healthy” and therefore, “unhealthy” – fitter, happier, more productive, etc.-, the patient always requests a change (or to restore a previous state) to the physician. Like during a thermodynamic reaction, some energy is lost in medical practice due to Entropy; this wasted energy that is related to unsuccessful/unordered reactions should be seen as all failures during this change of state (ineffective or inappropriate treatment, complication, side effect, social consequence, etc.). Also, as Entropy is linked to the notions of decay, chaos, and the unlikely possibility that occurs, it facilitates the understanding of ineffectiveness in Medicine: any unspontaneous change requires energy, the improbable will occur and, at the very end, medical practice fights against a natural and inexorable process towards death. Finally, Entropy may affect a minor part of the system to dysfunction with major consequences to the overall system; in terms of health, an accessory but impaired function might cause a chain reaction leading to a life-threatening condition. Furthermore, this dysfunction and conservation of energy (conservatism in most fields) might result in a lack of advances in medicine (including progress). Two distinct origins for this wasted energy might be recognized. First, from the individual standpoint, the treatment is not always successful, and a part of the energy, beliefs, and hope that the patient has invested in medical practice (and in doctors) will be lost. Then, from a collective standpoint, a part of the energy and means (money, time) involved in the management of patients will be lost.

Research in Medical Ethics analyzes this lost energy: the lost energy from the individual standpoint is individual ethics; the lost energy from the collective standpoint is public health. Furthermore, Ethics has to evaluate this wasted energy and compare it to the reaction, in order to assess if the risks are acceptable considering the benefits, from the individual and collective standpoint. This analysis is the pragmatic translation of the quest for meaning in medical practice; without criticism of this wasted energy, everything would be allowed regardless of the patient’s wish, benefits, and complications with or without treatment. Therefore, Entropy justifies research in Medical Ethics.

## Data Availability

Not applicable.

